# Exploring the Effect of Attachment Styles and Winning or Losing a Status Contest on Testosterone Levels

**DOI:** 10.3389/fpsyg.2018.01051

**Published:** 2018-07-17

**Authors:** Willem J. Verbeke, Frank Belschak, Tsachi Ein-Dor, Richard P. Bagozzi, Michaéla Schippers

**Affiliations:** ^1^Erasmus School of Economics, Erasmus University Rotterdam, Rotterdam, Netherlands; ^2^School of Economics and Management, University of Amsterdam, Amsterdam, Netherlands; ^3^Interdisciplinary Center Herzliya, Herzliya, Israel; ^4^Stephen M. Ross School of Business, University of Michigan, Ann Arbor, MI, United States; ^5^Rotterdam School of Management, Erasmus University Rotterdam, Rotterdam, Netherlands

**Keywords:** status games, testosterone, authentic pride, avoidant attachment style, anxious attachment style

## Abstract

A person’s ability to form relationships and seek and attain social status affects their chances of survival. We study how anxious and avoidant-attachment styles and subsequent winning or losing affects the testosterone (T) levels of team members playing two status contests. The first is a management game played by teams striving to earn the most profits. Winners and losers emerge due to the cognitive endeavor of the players, which provokes intense status dynamics. Avoidant-attached winners do not show higher T levels whereas anxious-attached winners do. The second is an economic game which is rigged and favors some teams to become richer than others; teams have the option though to trade with each other and reduce the self-perpetuating rich-poor dynamics embedded in the game. Besides attachment styles, we here also explore how authentic pride as a self-conscious emotion affects team members’ T levels as players trade with others to create more fairness. As in the first status contest, players’ T levels are not significantly affected by their avoidant attachment style, neither as a main effect nor in interaction with winning or losing the game. However, similar to the first game, players’ anxious attachment style affects their T levels: anxious-attached players generate significantly higher T levels when winning the game, but only when experiencing high authentic pride during the game. In short, the moderating effects of attachment style on winners’ T levels are partly replicated in both status games which allows us to better understand the functioning of working models of attachment styles during and after status contests and gives us a better understanding of working models of attachment styles in general.

## Introduction

A person’s ability to form relationships with conspecifics influences their chances of survival due to the effect this ability has on mental and physical health (Cacioppo et al., 2003). Attachment styles are self-relevant internal working models that encode the expectation and evaluation of care and acceptance by significant others in the case of need or stress (Mikulincer and Shaver, 2007; Vrtička and Vuilleumier, 2012). An attachment style is known to affect how people relate to each other and has therefore been the focus of much interest (e.g., Mikulincer and Shaver, 2009). Researchers distinguish between secure and insecure attachment styles, with the latter subdivided into anxious and avoidant-attachment styles (Mikulincer and Shaver, 2007).

The ability to seek and attain social status is another key element that affects a person’s chances of survival because attaining a higher social status compared to other conspecifics generally offers greater access to resources (Mazur et al., 1992; Salvador and Costa, 2009). However, little is known about the relationship between attachment style and seeking social status. The scarce literature indicates that these relationships are not clear cut, not only because of the complex operation of attachment working models in competitive contexts but also due to the differentiation of such contexts in which the working models are activated. Turan et al. (2014) show that avoidant as opposed to anxious-attached people tend to be dominant, cold and disconnected from others. In addition, avoidant-attached people also have higher basal testosterone (T) levels. In a personal relationship context, McDermott et al. (2017) show that both anxious and avoidant-attached students show dominant orientations. For anxious-attached people, partners who are pulling away from them might provoke frustration and so they might seek to draw their partner closer. Meanwhile, avoidant-attached people experience partners getting too close as threatening and thus might seek to maintain emotional distance. From there, people with both types of attachment styles use different means of psychological aggression. Using a priming experiment, Bartz and Lydon (2004) show that people primed with an anxious-ambivalent relationship increase the accessibility of agency because with a threat to their self, a shift in their working self-concept compensatory self-enhancement occurs. However, priming avoidant relationships did not provoke this effect. Note the heterogeneity of the different contexts and the clear lack of research into a context or situation where people’s status in a group is at stake, such as in status games, which might provoke specific working model dynamics and thus provide us with closer insights into the role of attachment styles when people engage in status competition.

Investigating this latter question is of interest because seeking proximity in the case of need and seeking status are both antagonistic social behaviors which people need to accomplish for survival. Yet, for some people this behavior might evoke goal incongruence or conflict, motivating them to pursue one above the other. This conflict might be especially salient in people with an anxious-attachment style as they tend to be communal as opposed to agentic (e.g., Bartz and Lydon, 2004), which would be an intuitive assumption at least.

Here we explore how attachment styles affect the participation of team members in competitive contests that are conceived as environments where people seek to gain status at the cost of others. Concretely, status implies a negative externality: an increase in one team’s relative status means a decrease in the relative status of the others (Heffetz and Frank, 2009). We are especially interested in how human T levels are affected when people win or lose a status contest and study how T levels are affected by a team member’s anxious or avoidant-attachment style. Intuitively, team members who win a status contest should have higher T levels (e.g., Geniole et al., 2017) and the team members who score high on avoidant-attachment style as opposed to those who score high on anxious-attachment style should have higher T levels when winning the status contest because they are agentic and dominant (e.g., Turan et al., 2014). However, these intuitive relationships might not occur for two reasons: (a) the dynamics of attachment working models might be adjusted when people experience a threat to their self, which occurs in status games (e.g., Bartz and Lydon, 2004); and (b) differences in the design of the contest – contextual variables – affect how status is attained (e.g., Geniole et al., 2017). To our knowledge this is the first study to focus on the relationship between people with different scores on attachment styles and T levels when winning or losing a status contest.

In this paper, we first briefly review the literature on status games and attachment theory and explore two different views on how attachment styles could affect T levels when winning versus losing status games. We introduce two games which vary in how they let team members attain their winning or losing position. The first study presents hypotheses on how attachment styles affect T levels when winning or losing a status game. The second study adds new moderating variables, namely authentic pride which is a self-conscious emotion that might be more or less activated in combination with attachment styles’ working models thus affecting T levels when winning or losing. We then discuss how the overall findings allow us to better understand how avoidant versus anxious-attached team members operate in status contests and how their attachment styles affect T levels when they win or lose. We conclude by discussing the limitations of our study and present ideas for future research.

### Status Contests and T Levels

Status contests and T levels are well studied in the literature. T is perceived as a social hormone which plays a role in regulating social relationships among conspecifics (Bos et al., 2012). The literature mentions two ultimate explanations as to why people seek to attain status (called status goals or motivations). (1) The challenge hypothesis, which originates from studies on birds’ T concentration fluctuations, depending on non-breeding versus breeding seasons and where birds’ T levels rise during breeding season is functionally linked to initiation of spermatogenesis. T levels rise even more during male-male competitions; that is, when both seek to attract a female or to gain territory (Wingfield et al., 1990; Archer, 2006). Importantly, homologous behavior and T level dynamics are also found in humans. When male and female humans are confronted with a threat to their status there is a rise in T levels that prepares them against potential status loss. (2) The winner (or biosocial status) hypothesis proposes that depending on the outcome of a status contest between competitive animals (including homo sapiens), where one is the winner and the other the loser, T levels rise in the animal that wins (Mazur, 1985; Mazur and Booth, 1998). Both explanations are closely related because status game winners tend to defend their winning position, and challenging their status again evokes intense T pulses; hence this is called the winner-challenge effect (e.g., Eisenegger et al., 2011; Fuxjager et al., 2016, p. 2). Proximal explanations for seeking and defending social status focus on the reciprocally related endocrine and psychological processes that are activated in such cases (e.g., Cacioppo and Berntson, 1992). In general, there is a consensus that human winners, whether alone or in a group (e.g., football team), have higher T levels and this level is higher in sports competition where spectators are present which is not the case in laboratory conditions (Geniole et al., 2017). The two games in this study are part of a university class assignment. The students are colleagues or friends who have known each other for at least 1 year. Hence these games do not mimic laboratory conditions but sports competitions in which a person’s status is publicly known.

### Attachment Systems and Attachment Styles

Formed during early life interaction with caretakers, attachment styles are trait-like dispositions that shape the innate attachment system. This is a biological goal-oriented system that motivates people to seek proximity with attachment figures in times of need, such as stressful situations (Mikulincer and Shaver, 2009; Beckes et al., 2014). Being close to attachment figures such as parents brings about a quiescent state in the child, with soothing feelings of warmth and calmness that off-load the stress onto the attachment figure and permit the child to broaden and build on their positive emotions (Beckes and Coan, 2015). The cumulative effect is that the child finds a secure base from where it can safely explore its own social environment or connect with other people (Fredrickson and Losada, 2005).

The attachment system is reflected in “working models of psychological mechanisms.” In this regard, Bartz et al. (2015) propose that attachment system activation comprises two main psychological processes: (1) expectations, whether specific people can be relied on to provide care and attention or are trustworthy in the event of need; and (2) the value or liking component which is the worth a person places on care, attention, or trust given by another person. Important for our study are different theoretical issues around attachment theory. First, working models can be perceived as chronic, imprinted mechanisms especially due to interaction with caretakers (the mother especially) and they are measurable with attachment scales. Second, these working models are context-sensitive, meaning that a person can activate multiple working models (avoidant or anxious) depending on the significant others or attachment figures being activated in their memory. They can also activate different working model dynamics, depending on the social context; e.g., when threatened in a competitive situation, self-compensatory self-enhancement can become part of the working model dynamics. Here we focus on chronic attachment styles and assume that they are formed at an early age; hence they operate as traits.

Attachment theory distinguishes three main attachment styles: avoidant, anxious, or secure. It has been argued that persons with a secure style score low on both anxiety and avoidance (e.g., Mikulincer and Shaver, 2007), hence for the purposes of this paper, we describe only avoidant and anxious-attachment styles and focus on working model dynamics.

Speaking figuratively, the avoidant-attachment style causes people to *deactivate* their attachment system in the case of need. When caretakers do not respond to a young child’s cry for help or do not provide the desired proximity (the default mode of the attachment system), the child develops low expectations about the help they are likely to get from others, and from there they come to place less value on attention or seek care from others less often. They end up developing an avoidant-attachment style, characterized by not expecting to co-regulate their stress with others very often. People with an avoidant-attached style appear self-reliant, seem proud of their self-reliance, have high self-esteem and higher baseline T levels, *yet* also remain largely indifferent to social support, criticism, or appreciation by other people (e.g., Mikulincer and Shaver, 2007).

Anxious-attached people also seek to down-regulate stress activation through *hyperactivation* of their attachment system. This occurs when caretakers respond to proximity seeking (default mode) inconsistently or become intrusive; children cannot learn to appraise others as reliable caretakers despite their desire to seek proximity and learn that care given by caretakers is unpredictable. In turn, this uncertainty only magnifies their desire for proximity, which makes them prone to expect that others will abandon them and thus creates a vicious expectancy-valuation cycle entailing fear of rejection or abandonment, and results in over-activation of the need for attachment. They end up remaining especially sensitive to signals of potential threat yet have a strong desire for proximity which makes attachment figures uneasy. Anxious-attached people develop low esteem, become over dependent, and are vulnerable to more rejection *despite* their high desire for social recognition (e.g., Mikulincer and Shaver, 2007).

### Two Status Games

To see if participants in a team status contest have higher T levels when winning, we use samples collected from two status games. Both games are described in detail in the Section “Methods” below. In study 1, team members exert control over winning or losing the contest through their own personal or collaborative cognitive efforts. The cognitive efforts reflect the professional/educational ambition of team members to become a company manager (clear and concrete status goal). During the game their own reputation is on the line, which we assume increases a conscious awareness of both their fear of losing and their desire to win. In this game the opposing teams start from equipollent positions, and that sets off intense competitive dynamics (e.g., from winning to a losing position and possibly back to a winning position) which might arouse team members. “Winner” or “loser” become clear social status signals which prime the T system both before the game (given clear indications that they can either win or lose) and after the game if they win or lose (given that winners are announced in public, thus placing losers in a less desirable position for them).

Study 2 is an economic game which makes team members rich versus poor. However, the players are allowed to some extent to reduce (or not) the gap in wealth which primes their economic status and compassion with the poor team members. The players (recruited from university students) are placed in three teams who are each given their own bag of coins. The bags contain an unequal distribution of coins representing differences in value indicated by three colors. These differences are such that the team members are part of the rich, middle and poor group (the latter two are called the losing groups). With each round, as they pick coins from the bag, team members soon realize that their richer versus poorer position is self-escalating. However, during the game each individual team member can choose to exchange coins according to specific rules: colors have to be different so that the richer team members give up some of their wealth to poorer team members. Alternatively, the richer teams can change the rules to favor either the poor team members or themselves. Although this game ultimately has winners or losers, compared to the first game, winning does not come from mere cognitive effort or battling it out. It depends on a mixture of luck, at being placed in a winning versus losing position (rigged) and a willingness to create more fairness between the teams, which we assume affects whether players experience authentic pride when winning the game. In contrast to study 1, although rich team members enjoy becoming richer (winning), this game also invokes breaking the self-escalation between rich and poor team members.

## Study 1

### Hypotheses

Teams start as equals and then battle for the winning position using their business skills and ability to think strategically to attain the win. The competitive context is transparent: the team that earns the highest financial return wins and from here we consider how the status game context might activate the psychological processes of the attachment working model. But given the scarce literature on the effect of competitive contexts on attachment working models, we present these hypotheses as exploratory.

Avoidant-attached team members value recognition or appreciation by others less than the low avoidant or anxious-attached members do (Vrtička and Vuilleumier, 2012). This is known as hypoactivation or deactivation of the attachment system. Consequently, they may not be that sensitive to either lower or higher status. Given their self-reliance (Mikulincer and Shaver, 2009), they should have less need to attain a dominant position (which always comes at the cost of others). Even if they try to win (it is their responsibility as a team member, and besides engagement in the game, is a course requirement of their study program), their T levels will not spike because their T system is not aroused by victory *over* others. There is another hypothesis, however: as avoidant people are dominant and agentic (Turan et al., 2014) and as other people might over take their status they will undertake extra efforts to remain dominant, hence their T levels will rise when they win. We explore two alternative plausible hypotheses:

*Hypothesis 1a:* When avoidant-attached players belong to the winning team of a status contest with clear winners and losers their T levels will remain low.

*Hypothesis 1b:* When avoidant-attached players belong to the winning team of a status contest with clear winners and losers their T levels will rise.

For anxious-attached team members, known to hyperactivate their attachment system, engagement in the status game will activate their attachment working model process (Mikulincer and Shaver, 2007). Anxious-attached team members place high value on winning due to their desire for social recognition and acceptance. Such high expectations, however, also amplify their awareness that they could lose or be perceived as a loser by team members, which puts greater psychological pressure on them compared to avoidant-attached or low anxious-attached people (Bartz et al., 2015). If they win, this will bring a sense of relief and excitement, as their desire to win is fulfilled and their expectation of losing disappears; hence their T levels rise. Another variation of an attachment style working model dynamic is that when anxious-attached people are confronted with a threat to their self, specifically status loss, they might engage in compensatory self-enhancement (Bartz and Lydon, 2004). They might see themselves and position themselves as defenders of the status of their team, or as potential winners, and so when winning, their T levels will rise. However, an opposite explanation is that when challenged, anxious-attached people hyperactivate the attachment system and the fear of losing becomes a dominant preoccupation of the working model; hence they might develop high fears and feel inferior or dependent (Bartz and Lydon, 2004). Due to the high fears, their cortisol level might rise, which is known to counteract the T system and thus even if they win, their T levels will remain low (e.g., Viau, 2002). We explore two plausible hypotheses:

*Hypothesis 2a:* When anxious-attached team members belong to the winning team of a status contest with clear winners and losers their T levels will rise sharply.

*Hypothesis 2b:* When anxious-attached team members belong to the winning team of a status contest with clear winners and losers their T levels will remain low.

### Methods

#### Participants

The games involved 119 Dutch students (71 males and 48 females; average age 25 years) who earned course credits for participating in our study as a component of their Master’s program in Business Economics. The Ethics Committee of the Erasmus Research Institute of Management (ERIM) at the Erasmus University, Rotterdam, approved the study and all participating students signed a consent letter. The students were given the game manual to prepare for the competition well in advance. Part of the game involved filling in a questionnaire that included such items as age, gender, and a short attachment styles measure. Requiring students to participate in this kind of research is similar to convenience sampling and grants limited control over gender, ambition, or other variables that characterize participants. This is why we created teams as follows: all participants were divided into groups that filled a session. On entering the session, the students were randomly assigned to a team. If students did not show up (due to illness or other obligations), then we reconfigured the teams, based on the available students. The absent students were invited to attend the next experimental session.

#### Measures

##### Biological assessments

Saliva samples were obtained with Sarstedt Salivette devices following standard salivary hormone collection procedures (Schultheiss and Stanton, 2009). The participants chewed on a synthetic swab at the onset of the game, and 25 min after disclosure of the final sales management team rankings. The samples were stored at -20°C until analyzed for T concentrations. Free T levels in saliva were analyzed with a commercially available ELISA kit (Demeditec Diagnostics, Kiel, Germany). The limit of detection was 34.7 pmol/L. The inter- and intra-assay coefficient of variation was less than 10%.

##### Psychological assessments

Attachment styles were measured with a 7-point disagree-agree items with “strongly” as end-points. These items were taken from Wei et al. (2007). Anxious attachment had two items and a Cronbach’s alpha of 0.74 (e.g., “My desire to be close sometimes scares people away”) while avoidant attachment had three items and a Cronbach’s alpha of 0.79 (e.g., “I try to avoid getting too close to others”). The factor structure of the attachment scales is usually found to be robust (see Wei et al., 2007), and in line with this, we found in a factor analysis two factors with eigenvalues larger than 1 to emerge from the attachment style items. On the first factor, the anxious attachment items loaded highly (factor loadings > 0.87); the avoidant attachment items loaded highly on the second factor (factor loadings > 0.80).

#### Procedure

##### The game

The sales management game is a computer simulation often used to train professional sales managers in how to make good business decisions (Patton, 1994). The game takes four real teams of managers (the participants) to manage virtual sales teams. The number of real managers per game varied from three to six players. Their task was to decide how to make their virtual sales team function most effectively. The team that made the highest cumulative earnings (profit) by the end of the game was the winner. Improving team effectivity required the managers to decide on whom they should hire from a list of candidates and deciding how to train, coach and reward their sales teams through the game. Their decisions were posted on the digital dashboard of the game. The names of the winning managers were posted on the classroom blackboard.

Each game contained four rounds, representing the quarters of a year. Gradually rising time constraints put management decision-making under pressure. The time decreased from 60 min in the first quarter, to 45, 30, and 20 min in the second, third, and fourth quarters, respectively. Between rounds, all four management teams received updates on how all the sales teams were doing so that they were aware of the dynamics of the status game.

The participants were reminded of the upcoming game 1 week before it started. They were asked to maintain their usual daily routine in terms of sport and sleep the day before and on the day of the game but not to smoke or eat chocolate or drink alcohol, tea, soft drinks, or coffee during the game, only water. They were fully briefed on the study and signed a consent form.

The game took place on different days but always between 11:30 AM and 5:00 PM to minimize the effects of circadian fluctuation on T levels (Touitou and Haus, 2000). Students were randomly assigned to one of four management teams, each of which had on average four or five players. At the start, the management teams listened to a detailed explanation of the aims and rules of the game, even though they were supposed to have read the game manual before the day of the game. During this session, they were told not to smoke, eat, or drink (except water). A half hour after the students entered the communal briefing room, we collected pre-contest saliva samples (T0), which measured their pre-game T levels. These pre-game levels were measured because they affect the level of T when winning the game (Casto and Edwards, 2016).

Each team was sent to their own game room, equipped with a computer on which to play the game. The researchers monitored the game and provided feedback from another room. At the end of each quarter, the management teams posted their strategy to a central computer that calculated the new results and produced a quarterly report, including data on the outcomes of decisions, such as sales revenue, company profits, and the names of the successful teams, plus a list of current staff (indicating successful or failed attempts to hire new salespeople). At the start of the subsequent rounds, the players were given progress reports, which included current rankings based on their own performance and that of the competing teams. This allowed them to make status comparisons.

At the end of the last round, the teams gathered again in the briefing room to learn the final rankings. They then waited an additional 25 min and kept on refraining from smoking, eating, and drinking. Finally, the post-game saliva sample was collected.

### Results

The analysis included only those players with completed survey and uncontaminated hormonal data, resulting in a total sample size of 119 respondents (71 male and 48 female). To test the impact of the game on the players’ hormonal responses (changes in T level), we computed *t*-tests comparing the T level before and after the game. The mean T level after the game was significantly lower than the mean T level before the game (pre-game mean = 185.59 pmol/L; post-game mean = 160.36 pmol/L; mean difference = 25.23 pmol/L, *t* = 4.54, *p* < 0.01). This finding is in line with the challenge hypothesis that indicates that T is a hormone that prepares people for battle and would lower in level when the challenge ends (Wingfield et al., 1990). The descriptives and intercorrelations of our variables of interest are presented in **Table 1** (upper part).

**Table 1:** Intercorrelations and descriptives of variables of interest.

	1	2	3	4	5	6
**Study 1**						
(1) Status						
(2) Anxious	0.18					
(3) Avoid	0.11	0.24**				
(4) Post T	0.11	-0.13	-0.09			
(5) Pre T	0.00	-0.09	-0.07	0.79**		
*M*	0.24	1.90	1.97	160.36	185.59	
*SD*	0.43	1.34	1.34	86.03	96.24	
**Study 2**						
(1) Status						
(2) Anxious	0.07					
(3) Avoid	-0.06	0.22*				
(4) Pride	0.38**	-0.04	-0.12			
(5) Post T	0.13	-0.00	-0.09	0.26*		
(6) Pre T	-0.00	-0.05	-0.11	0.25*	0.86**	
*M*	0.44	3.54	2.75	4.23	131.97	137.80
*SD*	0.50	1.03	1.30	1.32	52.78	52.32

^∗^*p < 0.05. ^∗∗^*p* < 0.01*.

To test the effect of this team status contest on players’ hormonal responses, we computed an analysis of covariance (ANCOVA) with the status achieved after the game (winner or loser) and the scores on anxious and avoidant-attachment style as independent variables and the post-game T level as the dependent variable. The analysis also controlled for the participants’ gender and pre-game T levels. Finally, we also controlled for team size (teams were not always equal in size). The explained variance of the variables and interactions on post-game T level is reflected in the partial eta-squared (η^2^). **Table 2** presents the results of the ANCOVA.

**Table 2:** Analysis of covariance (ANCOVA) results Study 1 (*n* = 119).

	*F*-value	*p*-value	η^2^	Parameter estimates	*SE*	95% CI
**Dependent variable: Testosterone level after status contest**						
Status (win/lose)	**4.430**	0.038	0.038	23.906	11.36	1.40; 46.41
Anxious attachment	1.569	0.213	0.014	4.636	3.70	-2.70; 11.97
Avoidant attachment	0.011	0.915	0.000	0.400	3.73	-6.99; 7.79
Pre-game T	**107.490**	0.000	0.490	0.625	0.06	0.51; 0.75
Gender	1.888	0.172	0.017	18.427	13.41	-8.14; 45.00
Team size	2.628	0.108	0.023	24.135	14.89	-5.37; 53.63
Status (win/lose)	**5.598**	0.020	0.048	-119.912	50.68	-220.35; -19.47
Anxious attachment	**8.555**	0.004	0.072	-3.144	4.11	-11.28; 5.00
Status ^∗^ Anxious	**13.627**	0.000	0.110	29.632	8.03	13.72; 45.54
Avoidant attachment	0.055	0.814	0.001	0.840	4.03	-7.15; 8.83
Status ^∗^ Avoidant	0.001	0.973	0.000	0.271	8.14	-15.86; 16.40
Pre-game T	**116.059**	0.000	0.513	0.617	0.06	0.50; 0.73
Gender	2.651	0.106	0.024	20.866	12.81	-4.53; 46.26
Team size	0.971	0.327	0.009	14.188	14.40	-14.35; 42.73

*Bolded values indicate significant values*.

The upper part of **Table 2** shows the main effects of our variables. Of our variables of interest, only status information (1 = win/0 = lose) was significantly related to post-game T level (*F* = 4.43, *p* < 0.05), while both anxious attachment (*F* = 1.57, n.s.) and avoidant attachment (*F* = 0.01, n.s.) were not. Of the control variables, only pre-game T level was significantly linked to post-game T level (*F* = 107.49, *p* < 0.01).

In a next step, we added interaction effects of status/anxious attachment and status/avoidant attachment to the analysis. As hypothesized, we found a significant interaction effect between status information and anxious-attachment style (*F* = 13.63, *p* < 0.01), but not for status information and avoidant-attachment style (*F* = 0.00, n.s.). Further, the pre-game T level had a significant effect on post-game T level (*F* = 116.06, *p* < 0.01); the other control variables were not significantly linked to post-game T level (gender: *F* = 2.65, n.s.; team size: *F* = 0.97, n.s.). The conditional effect of status on post-game T level was highly significant for participants high (plus 1 SD) on anxious attachment (*F* = 7.61; *p* < 0.01; *B* = 159.67) while it was not significant for those with low (minus 1 SD) anxious attachment (*F* = 3.18; n.s.; *B* = 80.15).

**FIGURE 1:**
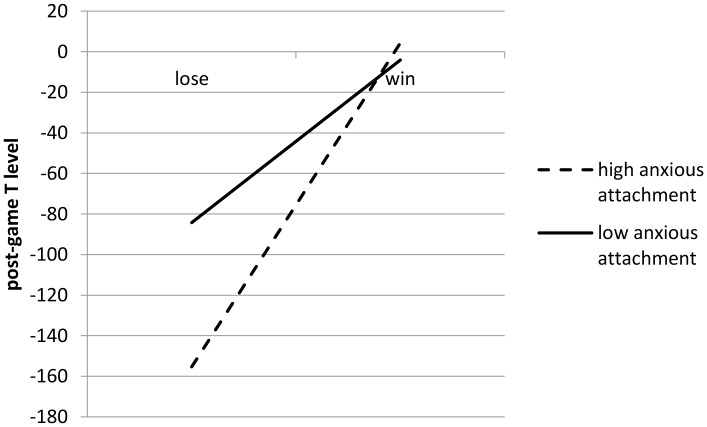
Interaction effect of status/anxious attachment on post-game T level for status game 1.

**Figure 1** shows the direction of the significant interaction effect between status information and anxious-attachment style on participants’ post-game T level based on the results of the ANCOVA. High anxious-attached winners showed a significantly higher post-game T level when compared to high anxious-attached individuals who lost the game. Low anxious-attached participants showed no significant difference in post-game T level as a reaction to status information.

### Discussion

Study 1 shows that avoidant-attached members do not show T spikes when winning the status game, which confirms exploratory hypothesis 1a and might indicate that they do not value social reward (winning) as they seek independence from others and are self-reliant. In other words, their attachment system remains deactivated. This finding does not substantiate hypothesis 1b that avoidant people are dominant and the status context motivates them to defend their dominant orientation hence when winning they will have higher T levels. Next, anxious-attached people have higher T levels on winning the game and this confirms exploratory hypothesis 2a. It proposes that anxious-attached people, whose attachment system is over-activated, become aware that the status game might threaten their self-esteem and thus acting as “a defender” against status loss might compensate for this threat. Further, if they win they might experience intense self-enhancement and thus develop higher T levels (Bartz and Lydon, 2004). In addition, winning would come as a relief due to their craving for recognition (Bartz et al., 2015). However, the assumption that a status threat during the game could overwhelm them (e.g., in terms of a higher cortisol level) and lead to a low T level seems not to be substantiated. To better understand this result we calculated the correlations between anxious/avoidant-attachment styles and pre-game T levels. As expected, these were not significant (anxious: *r* = -0.09, n.s.; avoidant: *r* = -0.07, n.s.) indicating that the effects of attachment styles (or the mechanisms of the working models of the attachment system) on T levels occurred during the game; e.g., tension during the game activated the attachment system of anxious-attached team members more intensely.

## Study 2

In addition to attachment styles, we explored psychological processes involving appraisal of the social environment when status is challenged or gained (Salvador and Costa, 2009, p. 165). In Study 2 we focused on self-conscious emotions “which are elicited when individuals direct attentional focus to the self, activating self-representations, and appraise an emotional eliciting event as relevant to those representations” (Tracy and Robins, 2007, p. 507). Specifically, we looked at authentic pride which may result from internal, unstable, and controllable causes (Tracy and Robins, 2007, p. 507). In general, feelings of authentic pride come with broader positive emotions (e.g., feeling positive about oneself compared to others in the future). The feelings are geared toward long-term attainment, maintenance of status, and promote status through relationship-oriented prosocial means (Tracy and Robins, 2007, p. 523). We explored whether attachment style and authentic pride affect T levels. Although rigged, the economic game a) still comes with status threats and b) it allows team members to behave prosocially, instilling authentic pride in some winning team members.

### Hypotheses

Players take part in a team status contest based on the economic distribution of wealth. Given that teams with access to more resources become richer and those with access to fewer resources become poorer, the game has a self-amplifying winning versus losing dynamic that is largely independent of effort. However, the players are invited to dampen the self-reinforcing mechanisms to bring some fairness into the division of resources between teams during the game. Would we thus find similar T patterns for winners as found in Study 1? Obviously, there would be many similarities (or replications) but also differences. First, both are status games where some team members are winners and others are losers. Second, however, winning or losing this game is rigged; no skill is involved. The team members have to draw coins of different values from a bag placed in front of each team. Each team’s bag has an unequal distribution of coins of lower or higher value, which makes drawing a valuable/less-valuable coin independent of effort and professional skill. However, a reasonable amount of literature shows that merely placing a person (or team) in a rigged winning position already raises T levels slightly (Newman et al., 2005; Schultheiss et al., 2005; Carré and Putnam, 2010).

As with the first study, we present two exploratory hypotheses why team members with avoidant or anxious-attachment styles will or will not have higher T levels. First, just like in the first game, avoidant team members experience low social reward when winning (Vrtička and Vuilleumier, 2012); hence, their T levels remain low even if they win. However, in a rigged game, for avoidant-attached team members (known to be dominant), winning would prime their dominant orientation or their agentic working model (Turan et al., 2014) hence they would experience high T levels. Therefore, we suggest the following two hypotheses.

*Hypothesis 3a:* When avoidant-attached team members belong to the winning team of a status contest that has no control over winning or losing, their T levels will remain low.

*Hypothesis 3b:* When avoidant-attached team members belong to the winning team of a status contest that has no control over winning or losing, their T levels will rise.

For anxious-attached team members with an over-activated attachment system, winning the rigged game will prime status goals; hence, we expect that their attachment system will be activated to a *certain extent* as no one wants to be stereotyped as poor (or a loser). However, due to the rigged set up of winners versus losers, T levels remain low. Nevertheless, as Bartz and Lydon (2004) argue, priming people with anxious-attachment words might also trigger agentic motivation; hence in this game a loss might activate identities of agency. In this case, they would celebrate victory with consequent rises in T levels. In addition, despite the rigged set up, for anxious team players winning might come as a relief from the mere thought of “if this game goes on like this, despite my efforts to swap coins, I will stay the poor person in my team” and T levels would rise when winning. Therefore, we explore these two possibilities:

*Hypothesis 4a:* When anxious-attached team members belong to the winning team of a status contest that has no control over winning or losing, their T levels will remain low.

*Hypothesis 4b:* When anxious-attached team members belong to the winning team of a status contest that has no control over winning or losing, their T levels will rise.

Winning also comes with a sense of authentic pride that might affect the T levels of the winners. Authentic pride not only helps a person enhance their own social status by informing them and their social group of individual success but also reinforces prosocial behavior (Tracy and Robins, 2007, p. 523). We expect that some team members might be more prone to experiencing authentic pride (Tracy and Robins, 2007). For instance, agreeableness is known to be positively related to authentic pride. Two reasons for the higher authentic pride when winning are proposed. First, as in the first game, for anxious-attached team members who are sensitive to negative emotions, facing a loss would serve as a compensating self-enhancement emotion and so, if they win they might have higher T levels (Bartz and Lydon, 2004). Second, this game might give team members several reasons to experience authentic pride, such as “even though I am on the winning team I had the chance to reduce the gap between rich and poor team members which makes me a fair winner.” We expect high anxious-attached team members as opposed to the low anxious-attached team members of the winning team who experience authentic pride to have higher T levels as they might be more prone to have compassion for the poor team members hence validating (not compensating for) the core self of their communal attachment working model (Bartz and Lydon, 2004). We do not expect avoidant-attached members to react as strongly to experiences of authentic pride as they are mostly indifferent to gaining status or peer recognition, let alone feel compassion for poor team members. Thus feelings of authentic pride would not amplify their T levels if they won the game (the reasoning applied in Study 1). Yet equally, as avoidant people are dominant and agentic, even in this rigged game they might feel authentic pride on winning.

*Hypothesis 5a:* When anxious-attached team members belong to the winning team of a status contest that has no control over winning or losing, and they feel authentic pride, this will cause their T level to rise.

*Hypothesis 5b:* When avoidant-attached team members belong to the winning team of a status contest that has no control over winning or losing, and they feel authentic pride, this will cause their T level to rise. Equally, due to their indifference to social reward this interaction might not take place.

### Method

#### Participants

A total of 105 students (54 male and 51 female, average age 23.3 years) earned credits for taking part in the experiment, which was a component of a MSc program in Economics and Business. They had different nationalities: 93 came from Europe, one from South America, ten from Asia and one from Africa. All participants signed the consent form; 95 provided full data on all variables of interest and were included in the final sample.

The Ethics Committee of the Erasmus Research Institute of Management (ERIM) at the Erasmus University, Rotterdam, approved the experiment and all participants signed a consent letter. Participants did not have to prepare for the experiment. During the first lecture of the core course, the students filled in a questionnaire, which included items such as age, gender, and their scores on attachment styles. They were randomly assigned to four groups of approximately 25 participants each. Later that week the students received an e-mail that informed them about their group and the starting time of their part of the experiment involving a game. The first two groups participated between 11:45 AM and 3:45 PM and the last two groups between 4:45 PM and 8:45 PM.

#### Measures

##### Biological assessments

We used the same method as in study 1 to obtain and analyze participants’ T concentrations.

##### Psychological assessments

The same attachment styles scale was used as in Study 1 (Wei et al., 2007). The anxious-attachment scale had five items and a Cronbach’s alpha of 0.71. The avoidant-attachment scale had five items and a Cronbach’s alpha of 0.88. The correlation between both scales was only moderate (*r* = 0.22, *p* < 0.05). Similar to Study 1, we again found in a factor analysis two factors for the attachment style items. On the first factor, the anxious attachment items loaded highly (factor loadings > 0.57); the avoidant attachment items loaded highly on the second factor (factor loadings > 0.76). Authentic pride was measured with five items taken from Tracy and Robins (2007). Cronbach’s alpha was high (alpha = 0.87). The full items are “I generally feel successful,” “… accomplished,” “… confident,” “I feel like I am achieving,” and “I feel like I have self-worth.”

#### Procedure

##### The game

The experiment used a business game designed by a Dutch company that specializes in serious games. On entering the game room students were invited to sit where they liked in one of three circles of chairs. Before the start, they were given a short explanation of the game and the game rules. The first round began with all the players drawing five coins from a bag sight unseen. This was followed by a round of trading. The trades have conditions: coins of equal value cannot be traded; negotiations start with a handshake and the two players concerned cannot let go of each other’s hand until they strike a deal. Players not open to trading coins must show this clearly by crossing their arms and remaining seated. If a player does not cross their arms, they have to accept a handshake and start negotiating.

After the first round, the meaning of the three circles of seats became clear. The first circle was for players with the lowest scores: the poor group. The second was for players with medium scores: the middle group. And the last one was for players with the highest scores: the rich group. During the game, the players noticed that it was hard to move from poor to richer groups and vice versa. This is because after the first round, players drew coins from a rigged bag that was assigned to the player’s group. This meant that the rich group had a greater chance of drawing valuable coins than the poorer groups, keeping the poor more poor, and making the rich richer. After all rounds were completed, the names of the three players with the highest scores were posted on the board, and each won €10.

Each game usually had five rounds. This number was not stated in the pre-game briefing to stop people from trying harder when they knew it was the last round. Trades were made in 5-min slots every round. Between rounds, all groups were given the chance to gain more points with a joker (wild card), worth ten points. Each group (rich, middle, and poor) received several jokers to share with their team members, but they had only 1 min to discuss the distribution.

In one of the later rounds, the rich group was allowed to change the rules in any way they liked. They could design three completely new rules for the remaining rounds. This was done to amplify the sense of unfairness and demonstrate how rigged the game was (poor players had no chance of reaching the top group, round after round.)

Just as in Study 1, 1 week before the start, participants were reminded of the upcoming experiment. They were asked to maintain their usual daily rhythms in terms of sport and sleep the day before and on the day of the game. They were asked to refrain from smoking, consuming alcohol, chocolate, tea, soft drinks, or coffee during the game, but they could drink water.

Upon arrival, students chose their seats at random. They were instructed not to smoke, eat, or drink (except water) and were given a bottle of water during the session. Immediately after the introduction, 30 min after entering the room and when rested, pre-contest saliva samples were collected. Then all participants gathered in the briefing room for a detailed explanation of the aims and rules of the game.

After the fifth round, all groups saw the final rankings. In the following 25 min, players filled in a questionnaire that measured experienced authentic pride using items from the scale by Tracy and Robins (2007). Having also refrained from smoking, eating, and drinking in this period they finally gave their post-game saliva samples.

### Results

Descriptives and intercorrelations of the variables of interest are presented in the lower part of **Table 1** (see Study 1 above).

As with Study 1, we tested the influence of the game on participants’ hormonal responses (changes in T level) by computing *t*-tests comparing the T level before and after the game. Again, participants’ mean T level after the game was significantly lower than their mean T level before the game (pre-game mean = 137.80 pmol/L; post-game mean = 131.97 pmol/L; mean difference = 5.83 pmol/L, *t* = 2.07, *p* < 0.05).

Next, we computed an ANCOVA with the status achieved after the game (i.e., 1 = winning or 0 = losing) and the players’ scores on anxious and avoidant-attachment style as independent variables and the post-game T level as the dependent variable. In addition, we included participants’ feelings of authentic pride after the game as an additional independent variable. We further controlled for participants’ pre-game T level as well as age and gender. **Table 3** shows the ANCOVA results. We found a significant main effect of status on participants’ post-game T level (*F* = 5.94, *p* < 0.05). The other independent variables showed no significant effects on post-game T level (anxious attachment: *F* = 1.490, n.s.; avoidant attachment: *F* = 0.00, n.s.; pride: *F* = 0.02, n.s.) (see upper part of **Table 3**).

**Table 3:** ANCOVA results for Study 2 (*n* = 95).

	*F*-value	*p*-value	η^2^	Parameter estimates	*SE*	95% CI
**Dependent variable: Post-game Testosterone level**						
Status (win/lose)	**5.940**	0.017	0.065	14.302	5.87	2.64; 25.97
Anxious attachment	1.490	0.226	0.017	3.355	2.75	-2.11; 8.82
Avoidant attachment	0.000	0.989	0.000	0.030	2.13	-4.21; 4.27
Authentic pride	0.016	0.901	0.000	-0.285	2.29	-4.83; 4.26
Pre-game T	**171.527**	0.000	0.666	0.809	0.06	0.69; 0.93
Gender	3.775	0.055	0.042	12.501	6.43	-0.29; 25.29
Age	3.034	0.085	0.034	3.050	1.75	-0.43; 6.53
Status (win/lose)	0.864	0.355	0.011	88.412	95.11	-100.95; 277.77
Anxious attachment	2.883	0.094	0.036	43.258	13.61	16.16; 70.36
Avoidant attachment	1.091	0.299	0.014	8.800	11.22	-13.53; 31.13
Authentic pride	1.445	0.233	0.018	23.294	10.11	3.17; 43.42
Status ^∗^ anxious	2.739	0.102	0.034	-39.361	23.78	-86.71; 7.99
Status ^∗^ avoidant	0.249	0.619	0.003	10.412	20.85	-31.11; 51.93
Status ^∗^ pride	1.337	0.251	0.017	-22.866	19.78	-62.24; 16.50
Anxious ^∗^ avoidant	2.785	0.099	0.034	-3.790	2.27	-8.31; 0.73
Pride ^∗^ anxious	1.497	0.225	0.019	-8.450	2.79	-14.00; -2.90
Pride ^∗^ avoidant	0.001	0.972	0.000	1.152	1.79	-2.41; 4.71
Status ^∗^ Anxious ^∗^ Pride	**4.505**	0.037	0.055	10.653	5.02	0.66; 20.65
Status ^∗^ Avoidant ^∗^ Pride	0.327	0.569	0.004	-2.459	4.30	-11.03; 6.11
Pre-game T	**180.565**	0.000	0.698	0.821	0.06	0.70; 0.94
Gender	3.627	0.061	0.044	12.263	6.44	-0.56; 25.08
Age	3.096	0.082	0.038	3.085	1.75	-0.41; 6.58

*Bolded values indicate significant values*.

These main effects were qualified by a significant three-way interaction between the conjectured status information, anxious attachment, and pride (*F* = 4.51, *p* < 0.05) (see lower part of **Table 3**). The corresponding three-way interaction for the avoidant-attachment style was non-significant (*F* = 0.33, n.s.), as were the effects of all two-way interactions. Finally, of the four control variables, only pre-game T had a significant effect on post-game T level (pre-game T level: *F* = 180.57, *p* < 0.01; gender: *F* = 3.63, n.s.; age: *F* = 3.10, n.s.). The conditional effect of status on post-game T level was only significant for individuals high on both anxious attachment and pride (i.e., both +1 SD from the mean), *F* = 4.51; *p* < 0.05; *B* = 173.28; it was not significant for other combinations of anxious attachment and pride (low/low: *F* = 0.32; n.s.; *B* = 32.32; low/high: *F* = 0.43; n.s.; *B* = 63.77; high/low: *F* = 0.98; n.s.; *B* = 84.28).

**FIGURE 2:**
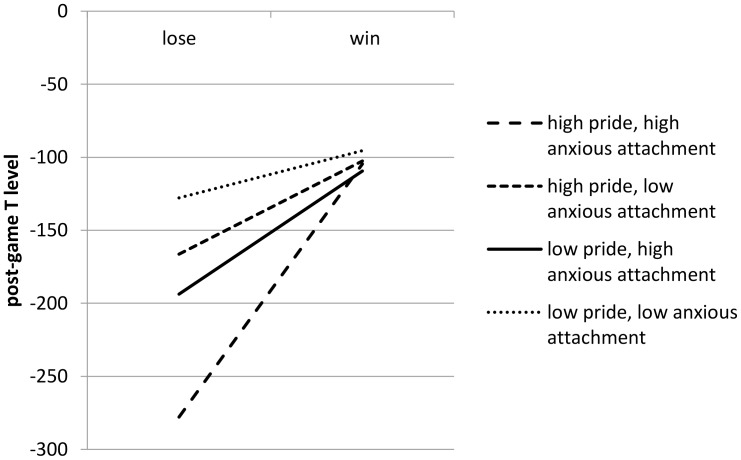
Interaction effect of status/anxious attachment/authentic pride on post-game T level for status game 2.

To facilitate interpretation, we plotted the significant interaction effect based on the slopes and intercepts. Specifically, we plotted the relationship between status (win/lose) and post-game T level for high and low values of the two moderators (**Figure 2**) (with high/low being defined as plus/minus 1 SD from the mean). The plot for participants with *high* feelings of authentic pride shows a pattern similar to the plot for the game in Study 1. Status information (winning versus losing) significantly influenced the T level for participants with high anxious attachment experiencing high levels of authentic pride. By contrast, status information was not significantly linked to the T level of participants with a low anxious attachment who experienced high levels of authentic pride. For those *low* on authentic pride, the plots show a different pattern: high anxious-attached people reacted no differently when winning compared to losing the game in terms of post-game T level. Similarly, those low on anxious attachment showed a non-significant increase in T level after winning compared to losing the game.

### Discussion

This self-reinforcing economic game – where rich teams became richer yet team members could reduce the gap between rich and poor teams – mostly replicated the findings of Study 1. First, winners high on avoidant-attachment style showed no higher T levels (hypothesis 3a), replicating Study 1. Second, there was no relationship between a team member’s score on anxious attachment and their T levels when winning. Thus the relationship between anxious attachment and higher T levels was not replicated, substantiating hypothesis 4a. Only winning team members with an anxious-attachment style and feeling of authentic pride had higher T levels. This might indicate that anxious-attached team members might experience status loss as a threat to their selves and might undertake a compensatory self-enhancing strategy, which shows up as authentic pride, which in turn elevates T levels (Bartz and Lydon, 2004). Alternatively, as we conjectured, because the rich team could help the poorer teams, a communal orientation might also have generated authentic pride. Note that winning team members with a high avoidant-attachment score and high on experience of authentic pride showed no increase in T level.

Finally, as with study 1 we tested whether the two attachment styles were significantly correlated with the pre-game T levels (anxious attached: *r* = -0.05, n.s.; avoidant attached: *r* = -0.11, n.s.). Again, they were not.

## General Discussion

Our research looked at whether T levels rise when team members win versus lose a status game, focusing on the moderating role of chronic anxious versus avoidant-attachment styles on T levels. We undertook this study because while attachment styles have been studied in the context of interpersonal relationships we were interested in how people’s attachment styles affect how they win or lose a competition, which for anxious-attached people especially *might* provoke feelings of conflict because they experience a communal rather than an agentic orientation; the latter of which is needed to engage in successful status battles.

The scarce research shows that specific competitive or conflicting contexts, which affect the dynamics in working models that characterize the attachment styles, result in dominating behavior. For instance, McDermott et al. (2017) show that when anxious-attached people notice that their attachment figure/partner is pulling away from them, they might use psychologically aggressive tactics; Bartz and Lydon (2004) show that priming people with anxious-ambivalent scenarios evokes a compensatory agentic identity. Note that these are personal relationship contexts and not actual status contests where one’s reputation comes at the cost of another. Given that this research project seeks to study the role of attachment styles on winning and losing a status game, which is different from interpersonal relational contexts, we presented exploratory hypotheses on how both anxious and avoidant-attachment styles affect team members’ T levels when winning a contest. We studied two status contests: in the first game, status had to be earned, while in the second the earned status was rigged. We were able to replicate some findings, taking into consideration the effect of contextual differences on how T levels vary when team members win versus lose a competition (Geniole et al., 2017).

It is important to emphasize that the two competitive games were different because in the first status game, where winning depended on effort, T levels were much higher than in the second study where earned status was rigged. In the first game, earned status was based on cognitive effort which made for clear winners versus losers. Thus participants might have taken this game very seriously or made it matter to their own professional identity. In the economic game, status won was more ambivalent because the game was clearly rigged and although it produced rich versus poor people, corrections could be made to the dynamics. Participants might have taken this game less seriously as it did not matter to their own professional identity. Indeed, T levels in the sales management game (Study 1) were higher (pre-game mean = 185.59 pmol/L; post-game mean = 160.36 pmol/L) than in the rigged game (pre-game mean = 137.80 pmol/L; post-game mean = 131.97 pmol/L), showing that the two games differed in the intensity of the endocrine and psychological dynamics they provoked. The first status game tested the business skills and ambitions of participants; hence it activated intensely salient status needs.

Note that replicating a study’s results does not necessarily mean that the experimental results should be similar; rather replication can also reveal boundary conditions. In addition, the status games shared a communality: both were salient for participant engagement because the games took place in exchange for credit and the names of the winners and losers were announced in class. In other words, all the students were aware of the importance of participation because they were going to see each other in class next week and thus knew that they had to defend their reputation. As Bartz and Lydon (2004, p. 1390) point out, attachment working models are sensitive to the specific relationships people have; in this case colleagues in class. Such contexts make the effect of attachment style on T level especially relevant. In addition, conforming to earlier research, we did not measure the T level scores of the teams but, similar to all the studies mentioned in Geniole et al. (2017), we studied the individual T level scores of the team members.

In a nutshell, this study discovered: speaking generally, due to their low need for social reward, avoidant-attached people did not have higher T levels on winning while anxious-attached people had higher T levels on winning, probably because the fear of losing either activated their agentic identity or the experience of relief from their fear of losing was responsible for the higher T levels. This was especially clear in Study 2 where people high on anxious attachment who felt authentic pride experienced higher T levels.

To our knowledge, these findings are unique in that we do not know of any other research that has studied the role of attachment styles in competitive contests. We believe that the findings present useful insights into the complex dynamics that attachment working models might produce.

First, although avoidant people are known to use a unique set of psychological violence in relationships when attachment figures become too close (McDermott et al., 2017) or are dominant and agentic in general (Turan et al., 2014), here we believe that our findings show that avoidant-attached people do not crave social reward and so do not have higher T levels when winning. In other words, they hypo-activate their attachment system when their status is at stake. We hope that other researchers will seek to explore these findings by adding similar variations in status games.

Second, while anxious-attached people hyperactivate their attachment system, which results in a communal rather than an agentic orientation, this might well evoke complex compensatory dynamics, as proposed by Bartz and Lydon (2004). Being seen to lose status in front of their class mates might evoke their agentic identity and prompt them to engage in intense efforts to come out of the game on the winning rather than the losing team. This compensatory agentic identity activation is especially salient in the rigged status game where authentic pride in combination with an anxious-attachment style affects higher T levels on winning.

### Further Interpretation of Our Findings

As anxious-attached winners have such high T levels, and as they are known to have high theory of mind expectations of other people’s intentions, such as whether or not others care about them, or knowing that others can challenge their status, our findings for anxious-attached members resonate with the findings of Fuxjager et al. (2016, p. 6) on T levels in competitive animal contexts. “Moreover, as Seyfarth and Cheney (2013) point out, we know little about the advantages of the ability to anticipate social changes, which presumably involves theory of mind to some extent (ability to attribute state of mind to others) that applies not only to affiliative behavior, but also, we speculate, to aspects of competitive behaviors in non-human animals.”

When thinking through the dynamics that the attachment working models could evoke, an important question is whether people high on attachment style might be better performers in general. Their heightened fear of losing or of being perceived as a loser might motivate them to work harder. For team members with low experience of authentic pride and high on anxious attachment losing or winning the game did not produce higher T levels. This might indicate that anxious attachment is associated with a readiness to keep worrying about something important. Such questions could be studied in future research.

### Limitations and Recommendations

We should of course mention the limitations of our study. Note that all the participants were Business Economics students who were following study trajectories lasting several years, who started the university careers by achieving high scores on entrance exams and who, in the university culture, constantly sought to build their social status. One of the consequences of calling on a population with this background is that the participants’ androgen receptors are already likely to be upregulated, which would allow them to respond more readily in any status fight; hence the observed high pre-game T levels. It is possible that people low on competitive experience might have lower pre-game T levels and thus we recommend exploring whether these findings can be replicated in other populations.

In addition, we need to note that the second game constitutes a more exploratory approach in which we aimed to investigate the effects of attachment styles in a different status game situation than in study 1. Consequently, the sample size of our second study was relatively low for the complex statistical analyses conducted. For instance, the *a priori* probability for detecting an existing significant three-way interaction effect of a small to medium size between anxious attachment, status, and authentic pride was less than 31% with our design. Future research should use more appropriate designs which, in case of Study 2, would mean to preferably use a sample of about three times our sample size in order to achieve a reasonable *a priori* chance (i.e., higher than 80%) for detecting the hypothesized interaction effect and to be able to draw conclusions from non-significant results. While we were lucky to find the interaction to be significant despite of the small *a priori* chance, a non-significant result would have been difficult to interpret and could be either because the effect is indeed non-significant or because our sample was too small to reliably detect such an effect.

We also need to acknowledge that data were nested (individuals playing the game in teams), but for anonymity reasons we were unable to identify individuals’ group membership and hence could not statistically control for such data nestedness. Nestedness of data is problematic as it causes statistical dependency in observations which can bias the standard errors associated with coefficients and may thus lead to wrong conclusions about the significance of relationships between variables, if not properly accounted for in the statistical analyses (e.g., Bliese and Hanges, 2004; Hox et al., 2017). Future research should therefore replicate our findings in studies explicitly designed to test our results and consider data nestedness and the related statistical dependencies.

Due to financial constraints, the Study 1 games were all played in the same time period, whereas Study 2 games were played both early and late in the afternoon. The timing (diurnal effect) might have affected the T levels. Note also that the size of the teams differed substantially in both studies. These factors might also have affected the findings in this paper by introducing different interpersonal dynamics. All the above speculations represent opportunities for future research.

## Ethics Statement

This study was reviewed and approved by the Ethics Committee of the Erasmus Research Institute of Management (ERIM) at the Erasmus University, Rotterdam. All research participants provided written and informed consent.

## Author Contributions

WV overall lead the paper, had set up and organized the experiments, lead in writing the paper and helped in computations. FB computed the data and worked on the method section. TE-D added insights from attachment theory to the paper. RB was involved in the writing of the paper. MS was involved in the writing of the paper.

## Conflict of Interest Statement

The authors declare that the research was conducted in the absence of any commercial or financial relationships that could be construed as a potential conflict of interest.
